# The effect of adding kinesiotaping versus pelvic floor exercise to conventional therapy in the management of post-colonoscopy coccydynia: a single-blind randomized controlled trial

**DOI:** 10.4314/ahs.v23i1.60

**Published:** 2023-03

**Authors:** Eladl Hadaya Mosaad, Aneis Yasser Mohamed, Attalla Asmaa Fawzy, Mohamady Heba Mohamed

**Affiliations:** 1 Cairo University Faculty of Physical Therapy, Department of physical therapy for Surgery; 2 Jouf University College of Applied Medical Science; 3 Cairo University Faculty of Physical Therapy, Department of Physical Therapy for Basic Sciences

**Keywords:** Coccydynia, colonoscopy, kinesiotaping, pelvic floor, tonic spasm, exercise

## Abstract

**Background:**

Coccydynia is a challenging disorder that is frequently managed conservatively.

**Objective:**

This study aimed to evaluate the efficacy of adding kinesiotaping versus pelvic floor exercise to conventional therapy in the management of post-colonoscopy coccydynia.

**Methods:**

Forty-two participants, aged 25–45 years, were randomly assigned to: the conventional therapy group (CT) receiving Piriformis and Iliopsoas muscle stretching exercise, clamshell exercise, and seat cushioning; the CT plus kinesiotaping group (CT-KT) receiving additional kinesiotaping; or the CT plus pelvic floor exercise (PFE) group (CT-PFE) executing additional PFE. All groups completed 4 weeks of training, 3 days a week. Pain intensity, assessed by the Pain Numeric Rating Scale (PNRS), and functional disability, evaluated by the Oswestry Disability Index (ODI), were estimated at baseline and after 4 weeks.

**Results:**

There were significant inter-group variations in PNRS and ODI, where (P = 0.0001) and (P = 0.03), respectively. Differences between experimental groups were noteworthy in terms of NPRS, where the major change was in favor of group CT-KT (P = 0.001). However, there was no significant difference between them regarding their impact on ODI.

**Conclusion:**

CT-KT is more effective than CT-PFE in reducing pain associated with coccydynia post-colonoscopy, but there is no difference in their effects on functional disability. CT-KT is therefore recommended as an alternative treatment option for post-colonoscopy coccydynia.

## Introduction

Colonoscopy is a common procedure used to diagnose and treat a wide variety of conditions^1^. While generally regarded as a safe procedure, colonoscopy complications as an invasive approach range from mild symptoms such as mild abdominal pain to more severe complications such as colonic perforation, cardiopulmonary arrest, or even death. Coccydynia is one of the complications that result from internal trauma to the coccyx or its surrounding^2^. Coccydynia is a non-radiating pain that is located in the coccyx or its vicinity and is exacerbated by sitting and transitional movements, with females five times more likely to be affected than males^3^. Related symptoms are dyspareunia and pain on defecation, which are assumed to be pelvic floor muscle manifestations^4^. Originally, Thiele^5^ proposed pelvic floor muscles as the key drivers of coccydynia by describing a vicious cycle in coccydynia development. However, following Thiele's original hypothesis, many reports have pointed out and verified the existence of pelvic floor muscle spasms and pelvic floor myofascial pain in patients with coccydynia 4,6,7.

Patients report “tailbone discomfort” that gets worse with extended sitting, leaning back while seated, prolonged standing, and rising from a seated position, as well as tenderness over the coccyx^8^. Patients experience a significant decrease in their quality of life^9^.

Pelvic floor exercises (PFE) were proposed as an effective modality. PFE involves training the muscles of the pelvic floor to contract and, much more importantly, to relax fully. The proposed mechanism is that the contraction-relaxation exercises minimize the muscle's resting tone and interrupt the spasm and pain cycle 10,11.

Surgical intervention via coccygectomy has been reported to relieve approximately 50% to 90% of symptoms. However, due to the unpredictable long-term effects and the risk of major complications, surgery is rarely performed, and non-surgical strategies remain the major treatment for coccydynia 12. Current literature provides several options for the conservative management of Coccydynia; non-steroidal anti-inflammatory drugs, ring-shaped cushions, Levator ani relaxation exercises, and gentle massage over the ligaments attached to the sacrococcygeal joint^8^,^13^. Physical therapy modalities include interferential current^14^, shortwave diathermy^15^, extracorporeal shock wave therapy^16^, stretching of the Piriformis and Iliopsoas muscles^17^, coccyx manipulation^18^, and kinesiotaping (KT)^19^.

KT is a therapeutic approach that has emerged recently as a viable alternative for treating different musculoskeletal and neuromuscular disabilities 20. KT can be used to restore muscle function, realign soft tissue, enhance lymphatic and vascular flow, decrease pain sensitivity 21, 22, and reduce muscle tension 23. Moreover, the use of KT can modify the pattern of muscle fiber recruitment 24. However, from a practical perspective, physiotherapists do not employ KT as an independent intervention but rather as an additional part.

Although clinical guidelines recommend the conservative management of coccydynia, most randomized trials from which these guidelines are derived have shown that, when used in isolation, these therapies provide only mild to moderate clinical advances. In addition, there is no distinction between the various exercise-based therapy approaches and the various manual therapy techniques 7, 8, 17. Given the limited clinical progress and the absence of a leading intervention, new approaches are being explored in a range of physiotherapy strategies to optimize treatment efficacy and enhance patient satisfaction. Therefore, the present study aimed to evaluate the efficacy of adding kinesiotaping versus PFE to conventional therapy (CT) in the management of post-colonoscopy coccydynia.

## Methods

This study was a randomized, single-blind (blinded assessor), controlled trial conducted at the physiotherapy clinic of Cairo University Hospitals during the period from January to December 2017. This trial was approved by the Faculty of Physiotherapy's Institutional Review Board, University of Cairo **[P.T. REC/012/002613]**, reported in the Clinical Trials database **[NCT04261647]**, and is in compliance with the CONSORT Guidelines. All participants were given a thorough explanation of the study's methods and goals, and they were asked to give informed legal consent to participate in the study and generalize the findings.

Participants were referred to our physiotherapy clinic by an orthopedist with a definite diagnosis of post-colonoscopy coccydynia. Patients were selected to be enrolled in the study upon fulfilling the following inclusion criteria: medically stable males and females were included, being at least 2 weeks post-colonoscopy and aged 25–45 years, with pain during defecation and difficulty in the cross-sitting position. Difficulty leaning back during a sitting position, tenderness over or around the coccyx. With some sort of pain during intercourse. The pain score on the numerical pain rating scale was not less than 6. Patients with serious spinal pathology (e.g., fracture, tumour, inflammatory and infectious diseases), pelvic surgery, herniation of the lumbosacral disc, skin disorders, genitourinary or gastrointestinal complaints were excluded.

Patients who met the study's inclusion criteria were randomly allocated to one of three groups: the conventional therapy group (CT) that received Piriformis and Iliopsoas muscle stretching exercises, clamshell exercise, and seat cushioning; the CT plus kinesiotaping group (CT-KT) who underwent kinesiotaping in addition to the CT; or the CT plus pelvic floor exercise group (CT-PFE) who received PFE in the form of reverse Kegel exercise in addition to the CT.

**Figure 1:**
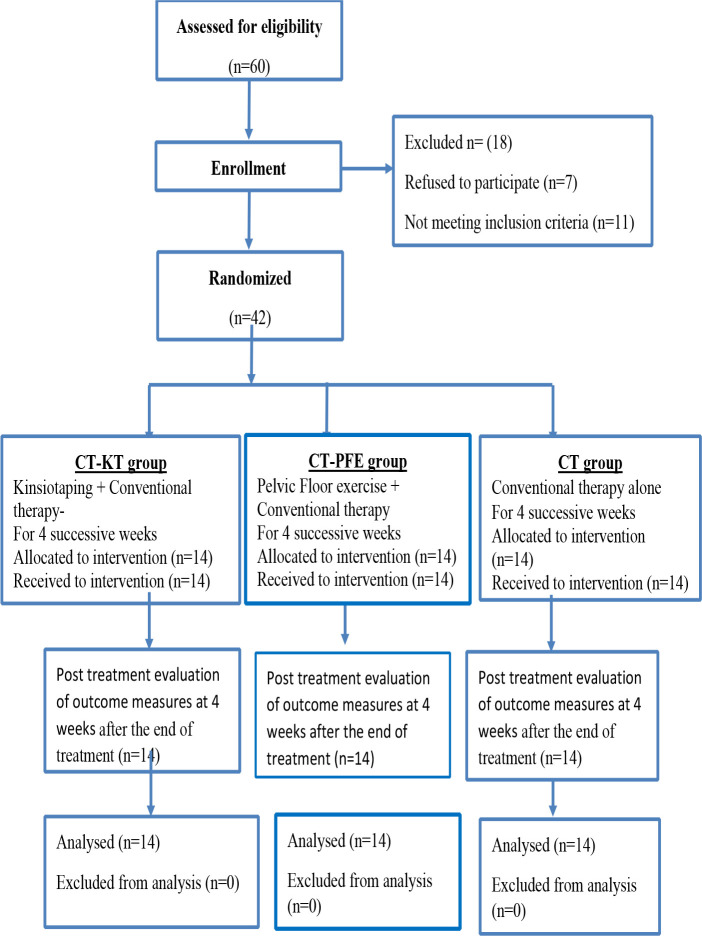
Flow diagram showing the progress of subjects at each stage of the clinical trial

Participants were randomly assigned using computer-generated block randomization. The allocation was done through the launch of an obscure envelope designed by an independent subject. Before commencing the intervention, the trial-supervising therapist opened the envelope and revealed the treatment of choice corresponding to the number in the envelope. Figure 1 provides a flow diagram of the study design.

### Assessment

Patients' characteristics were obtained using an evaluation form explicitly designed for this trial. This form included questions concerning the demographic and anthropometric features as well as the health status of the individual, such as drug usage, physical activity level, educational attainment, and history of coccydynia and its related-symptoms.

### Pain numeric rating scale

The Pain Numeric Rating Scale (PNRS) evaluates the pain intensity experienced by the patient using a scale of 11 points (extending from 0 to 10), with 0 representing “no pain” and 10 indicating “the worst pain imaginable”. The participants were advised to record the pain intensity rates 25.

### Functional disability

The functional disability was evaluated with the Oswestry Disability Index (ODI). It is a self-reported questionnaire of 10 questions reflecting the participants' functional activities (self-care, sitting, standing, walking, sleeping, lifting, sexual activity, traveling, social life, and work). For every item, there are six possible answers, ranging from no disability (0) to complete disability 5. The ODI is scored between 0 and 50, with higher scores suggesting greater disability 26.

All outcomes were obtained by an assessor blinded to patients' treatment allocation at baseline and 4 weeks post-intervention.

### Interventions

#### Conventional therapy

Over 4 weeks, all patients received CT three times a week, consisting of Piriformis and Iliopsoas muscle stretching exercises, clamshell exercise, and seat cushioning.

### Piriformis stretching

As starting, to stretch the right Piriformis, the patient lies supine and the right femur is flexed and laterally rotated such that the right ankle rests on the posterior aspect of the distal left thigh. From this position, the left hip is flexed until tension is perceived in the right buttock. Passive stretching is provided for a 30 second hold. The same procedures were carried out for the left Piriformis^27^.

### Iliopsoas stretching

Stretching was executed in crook lying, with both lower limbs hanging at the end of the table, so that the stretched hip could be reached beyond neutral. The opposite hip and knee were bent toward the patient's chest while stretching to support the pelvis and spine. The hip to be stretched, moved into extension or hyperextension while stabilizing the opposite leg by applying downward pressure to the anterior aspect of the distal thigh. The knee could be extended so that the two-joint rectus femoris would not limit the range. Stretching was kept for 30 seconds. The same procedures were applied on the other side 27.

### Clamshell exercise

Participants assumed a side-lying position with both hips flexed at 45°, knees bent at 90°, and keeping their heels and the 1st metatarsal head together. The subjects separated their knees and rotated the top leg upward as much as possible. Clamshells were performed on both sides for three sets of 10 repetitions 28.

### Pelvic floor relaxation exercise

The reverse Kegel exercise was used to alleviate the tension in the pelvic floor muscles. While the patient is sitting comfortably, begin by gently contracting the pelvic floor as he would to interrupt the stream while urinating, taking care not to use other muscles in the abdomen, legs, or buttocks. The patient is then asked to breathe deeply and bring his mind to the pelvic floor, so that he can feel his muscles relax and fall down while inhaling. Hold the reverse Kegel for 5 seconds, then release the same time for three sets of 10 repetitions 27.

### kinesiotaping technique

Before kinesiotaping, the lower back skin was shaved to avoid the unpleasant sensation associated with the removal and replacement of the tape. The skin was cleansed by swabs of alcohol to combat bacterial growth and invasion of the skin that triggers cellulitis. Kinesiotaping was executed as patients assumed a stooping posture and leaning forward; seven 2.5-cm wide I-shaped strips (Albuquerque, NM, United States) were secured to the base of the tail bone and pulled up to the horizontal line, spreading in a fan shape between both PSISs. The patient was then asked to take a standing position, and the tape wrinkles were examined to ensure proper application. The tape was replaced every 72 hours 29.

### Outcome measures

The primary outcome was the change in pain intensity assessed by PNRS, whereas the secondary outcome was the change in functional disability measured by ODI. All measures were estimated at baseline and after 4 weeks of intervention.

### Statistical analysis

Before final analysis, data were assessed for the assumptions of normality and homogeneity of variance, and revealed no violations for any of the dependent variables, as assessed by Shapiro-Wilk test and Levine's test, respectively. Consequently, for all groups, descriptive statistics were estimated at two-time intervals: baseline and following four weeks of intervention. The Paired t-test was used to analyse within-group differences, and the analysis of variance (ANOVA) was used to differentiate between groups with regard to the selected parameters pre- and post-intervention. To estimate the average impact of the intervention, Cohen's d was used to assess effect sizes **30**. The statistical analysis was carried out using SPSS software for Windows, version 21.0 (Chicago, IL, United States). The significance level was set at P ≤ 0.05.

## Results

Figure 1 demonstrates the flow of participants from recruitment to follow-up; 42 (70%) of the 60 patients assessed for eligibility were randomly assigned to groups. In each group, all patients adhered to the intervention and achieved follow-up. No side-effects were documented by any patient due to the intervention.

Data review revealed no significant variations between groups in terms of demographic and clinical characteristics pre-intervention (P>0.05), as demonstrated in Table 1. For PNRS and ODI, there were significant changes in the three groups post-intervention, with more enhancements in the two experimental groups by a change of (↓68.48%, ↓42.33%, and ↓21.56%) and (↓85.21%, ↓86.20%, and ↓46.53%), for CT-KT, CT-PFE, and CT, respectively. Between-group differences were noteworthy where (P = 0.0001) and (P = 0.03), respectively. The results are illustrated in Table 2.

**Table 1:** patients' demographic and clinical characteristics (Mean ± SD)

Variables	CT-KT Group (N=14)	CT-PFE group (N=14)	CT group (N=14)	P-value
Age (years)	44.92 ± 6.20	43.62 ± 5.00	45.78 ± 5.38	0.594
Height (cm)	155.40 ± 6.67	157.30 ± 5.75	159.62 ± 6.65	0.113
Weight (kg)	68.67 ± 6.73	67.55 ± 6.54	66.90 ± 5.95	0.251
BMI (Kg /m^2^)	27.28 ± 4.95	28.14 ± 4.78	26.98 ± 5.74	0.652
Gender (female)	65.31%	69%	70%	0.450 ^a^

CT= conventional therapy; KT= kinesiotaping; PFE= pelvic floor exercise; SD = Standard Deviation; a = the value is calculated using the Kruskal-Wallis's test; BMI = Body Mass Index; Level of significance at P ≤ 0.05.

**Table 2:** Outcome measures for PNRS and ODI at baseline and after 4 weeks of intervention

Variable	CT-KT Group mean ± SD	CT-PFE group mean ± SD	CT group mean ± SD	*P*-value			
					CT-KT Vs CT-PF	CT-KT Vs CT	CT-PFE Vs CT
PNRS							
Pre-intervention	7.14 ± 1.40	7.18 ± 1.46	7.28 ± 1.26	0.950			
Post-intervention	2.28 ± 1.13	4.14 ± 1.61	5.71 ± 1.13	0.0001*	0.001*	0.0001*	0.003*
% Of Change	↓ 68.48	↓42.33	↓21.56				
*P*-value	0.0001*	0.0001*	0.0001*				
Effect size (Cohen's d)	3.03	1.12					
ODI							
Pre-intervention	32.07 ± 16.20	34.64 ± 14.40	35.64 ± 13.10	0.802			
Post-intervention	5.07 ±4.75	4.78 ± 3.19	12.64 ± 10.13	0.03*	0.908	0.029*	0.022*
% Of Change	↓85.21	↓86.20	↓64.53				
*P*-value	0.0001*	0.0001*	0.0001*				
Effect size (Cohen's d)	0.95	1.04					

CT= convent ional therapy; KT= kinesiotaping; PFE= pelvic floor exercise; SD = Standard Deviation; PNRS = pain numeric rating scale; ODI = Oswestry Disability Index; Level of significance at P ≤ 0.05

* Significant

Considering the differential effects of the three groups on NPRS, multiple pairwise comparison analysis showed a substantial difference between the three groups with P (0.001, 0.0001, and 0.003), respectively. The significant enhancement was in favour of group CT-KT with an effect size of (3.03) compared to groups CT-PFE and CT, and in favour of group CT-PFE with an effect size of (1.12) compared to group CT. Regarding their effects on ODI, there was no significant difference between CT-KT and CT-PFE where P = 0.908. However, there was a significant difference between [CT-KT vs CT with an effect size of (0.95) and CT-PFE vs CT with an effect size of (1.04)], where P (0.029 and 0.022) respectively. Results are represented in Table 2.

## Discussion

The findings of the current trial showed that after 4 weeks of intervention, all groups reported substantial differences in PNRS and ODI relative to baseline, with more enhancements in the two experimental groups. Differences between the experimental groups were notable in terms of NPRS, where the major change was in favour of group CT-KT. Nevertheless, there was no difference between them with respect to their impact on the ODI. Pelvic muscles are 70% slow-twitch, striated skeletal muscles that include the muscles of the coccygeus and the muscle complex of the levator ani, including the puborectalis, pubococcygeus, and iliococcygeus. These muscles preserve the tone of the pelvic floor and protect the connective tissues from overload^31^.^32^.

Injuries to the coccyx or coccygeal joints, or where the underlying tissues were the seat of inflammation, any tightening of the muscles attached to the coccyx would induce the coccydynia's characteristic pain 32. Patel et al. highlighted the importance of over-activity and other anomalies that affect the pelvic floor musculature in patients with coccydynia^6^.

Pain is caused primarily by the tonic spasm of the pelvic floor muscles. Thiele^5^ described a vicious cycle of pelvic floor pain pathogenesis. After a triggering event, the muscles of the pelvic floor, the levator, and the coccygeus experience spasms. It should be acknowledged that muscle spasm, indeed, by itself, is a distressing disorder. Spasm of both levator parts induces forward and lateral coccygeus muscle traction. Unilateral coccygeal muscle contraction draws the coccyx to one side, and the pain tends to increase along with the co-existing pain or degeneration of the sacrococcygeal joint, leading to further spasm^5^.

The efficacy of KT for pain relief and functional disability enhancement has been reported in several studies^19^,^33^,^34^. KT has been proposed as an efficient method for enhancing blood and lymphatic circulation, realigning joints, and normalizing muscle tension^21^–^23^. Another potential mechanism is that the cutaneous stretch stimulation given by KT can interfere with the transmission of mechanical and painful stimuli, providing afferent stimuli that promote pain-inhibiting mechanisms^24^. Moreover, it is hypothesized that enhanced afferent feedback would activate the neuromuscular pathways^29^.

In a study conducted by Donec et al. that investigated the effect of KT on the post-operative pain of total knee replacement and discovered that KT, when combined with other rehabilitation modalities, had a pain-relieving effect^35^. The findings of their investigation matched those of another study by Imperatori et al., which used KT to reduce pain following lung cancer lobectomy. The authors concluded that KT is a safe and effective approach for reducing chest pain that complements oral analgesics, as evidenced by a one-point decrease in the visual analogue scale on the 5^th^ and 8^th^ post-operative days^36^.

Several types of manual therapies have been recommended for Coccydynia, including levator ani, coccygeus, and piriformis muscle massage^37^; joint mobilization^7^; or gentle manual levator ani muscle stretching^13^. Maigne and Chatellier contrasted these three types of manual therapy and reported success rates of 29.2, 16 and 32% with massage, mobilization and stretching approaches, respectively, after 6 months of follow-up^13^. Nevertheless, pelvic floor physical therapy emphasizing overactive pelvic floor muscle down-training is successful in treating even long-standing coccydynia^38^.

In terms of the effect of PFE on hypertonic muscles, our findings are consistent with those of Gentilcore-Saulnier et al., who examined women with induced vestibulodynia before the implementation of PFE and found that their superficial pelvic floor muscles had higher tonic surface electromyography activity relative to controls. After eight sessions of PFE, women with vestibulodynia had a decreased pelvic floor muscular reaction to pain, decreased pelvic floor muscle tone, and enhanced pelvic floor muscle capacity^39^.

The changes in patients undergoing reverse Kegel may be a function of the muscle length-tension relationship. Patients with overactivity have mechanically shortened muscles, thereby reducing the number of cross-links necessary to produce the desired force of contraction. By relaxing the pelvic floor and increasing the length of the muscle, there are further crosslinks to create a stronger and more efficient contraction. Overactive pelvic floor muscles often cause trigger points to develop. Returning the muscle to its optimal resting length can help reduce coccydynia pain while also increasing the intensity and synchronization of pelvic muscle contractions and hence functional ability^40^.

There are some potential drawbacks to the present trial. First, the relatively small sample size, although adequate to identify statistically meaningful differences between groups, However, it would be more advantageous to execute randomized trials with larger sample sizes to obtain more reliable results. Second, we used a convenience sample that may not be representative of the whole population of individuals with post-colonoscopy coccydynia, Furthermore, there are no follow-up details on the participants' health status, which would help us track the long-term effects of both interventions. Therefore, it would be beneficial to address this in future studies.

## Conclusion

CT-KT is more effective than CT-PFE in alleviating pain related to post-colonoscopy coccydynia. However, there is no difference in their effect on functional disability. Therefore, CT-KT is recommended for patients with post-colonoscopy coccydynia as an appropriate treatment option.
